# Topiramate Treatment Is Neuroprotective and Reduces Oligodendrocyte Loss after Cervical Spinal Cord Injury

**DOI:** 10.1371/journal.pone.0033519

**Published:** 2012-03-13

**Authors:** John C. Gensel, C. Amy Tovar, Jacqueline C. Bresnahan, Micheal S. Beattie

**Affiliations:** 1 Center for Brain and Spinal Cord Repair, Department of Neuroscience, The Ohio State University College of Medicine, Columbus, Ohio, United States of America; 2 Brain and Spinal Injury Center, Department of Neurological Surgery, University of California San Francisco, San Francisco, California, United States of America; Case Western Reserve University, United States of America

## Abstract

Excess glutamate release and associated neurotoxicity contributes to cell death after spinal cord injury (SCI). Indeed, delayed administration of glutamate receptor antagonists after SCI in rodents improves tissue sparing and functional recovery. Despite their therapeutic potential, most glutamate receptor antagonists have detrimental side effects and have largely failed clinical trials. Topiramate is an AMPA-specific, glutamate receptor antagonists that is FDA-approved to treat CNS disorders. In the current study we tested whether topiramate treatment is neuroprotective after cervical contusion injury in rats. We report that topiramate, delivered 15-minutes after SCI, increases tissue sparing and preserves oligodendrocytes and neurons when compared to vehicle treatment. In addition, topiramate is more effective than the AMPA-receptor antagonist, NBQX. To the best of our knowledge, this is the first report documenting a neuroprotective effect of topiramate treatment after spinal cord injury.

## Introduction

Tissue loss after spinal cord injury (SCI) is the result of primary physical damage and secondary apoptotic and necrotic cell death. Cytokines, neurotransmitters, and blood products released by the initial physical trauma create a toxic environment for neurons and oligodendrocytes. For example, levels of the pro-inflammatory cytokine, TNF-α, increase acutely after injury. Neurons, in response to high levels of TNF-α, traffic glutamate receptors to their cell membrane thereby becoming more sensitive to excitotoxic cell death [1]. TNF-α also increases the susceptibility of oligodendrocytes to excitoxic cells death [2]. Levels of pro-inflammatory cytokines and glutamate increase after injury coincident with neuron and oligodendrocyte loss [3].

Although extracellular levels of glutamate reach toxic levels shortly after SCI [4], [5], delayed administration of drugs that directly antagonize glutamate receptors reduces cell loss and improves motor recovery [6]. Even when delayed by 4 hrs after SCI, the alpha-amino-3-hydroxy-5-methyl-4-isoxazolepropionic acid (AMPA)/kainic acid (KA) glutamate receptor antagonist NBQX improves functional recovery and tissue sparing [7]. Unfortunately, this drug, and many other glutamate receptor antagonists, have detrimental side effects and have largely failed clinical trials [8]–[10].

In an effort to find clinically feasible approaches for limiting excitotoxic cell death after SCI we tested the neuroprotective potential of topiramate treatment in a model of cervical SCI. Topiramate is a potent AMPA receptor antagonist FDA approved for clinical treatment of epileptic seizures and migraines. Topiramate treatment is also neuroprotective and rescues oligodendrocytes in models of traumatic brain injury, stroke, epilepsy, and Periventricular leukomalacia [11]–[14]. We report that topiramate delivery 15 minutes after SCI increases tissue sparing, preserves oligodendrocytes, and saves motor neurons compared to vehicle and NBQX controls. To the best of our knowledge this is the first report examining the neuroprotective potential of topiramate in a SCI model.

## Methods

### Animals and Surgery

Fifty-two female, Long-Evans rats (Simonsen Laboratories, CA, USA) 75–85 days old and weighing an average of 236+/−2 grams were anesthetized with sodium pentobarbital (Nembutal, Abbott Laboratories, IL, USA; 45–60 mg/kg) and received prophylactic antibiotics (cefazolin; Ancef, Novation, LCC, TX, USA; 25 mg/kg). Body temperatures were monitored throughout the surgical procedure and maintained at 37+/−2°C. Cervical contusion injuries were performed as described in detail previously [15]. Briefly, a single level laminectomy was performed at C5 and right unilateral SCI produced using the MASCIS/NYU injury device [16], [17]. Two severities of injury were produced by dropping a 10-gram weight from a height of either 12.5 mm or 6.25 mm. The 12.5 mm weight drop damages >80% of the tissue ispilateral to the injury and is hereafter referred to as a severe injury [15]. The 6.25 mm weight drop damages ∼50% of the hemicord and is hereafter referred to as a moderate injury [15].

### Drug administration

The dura was opened immediately after SCI and the animal was placed in a stereotaxic frame. Twelve minutes and thirty seconds after contusion a 10 µl bevel-tipped Flexifil microsyringe (diameter = 180 um; World Precision Instruments, FL, USA) was lowered to a depth of 2.0 mm into the center of the contused area of the spinal cord using a micromanipulator. The spinal cord was allowed to acclimate to the syringe for 2.5 minutes before the solution was delivered. Starting 15 minutes after the contusion, 3 µl of one of three solutions was delivered over 10 minutes: 1) 8.9–9.3 mM NBQX (NBQX disodium salt; Tocris, MO, USA) dissolved in water; 2) 10 mM topiramate (Sigma, MO, USA) dissolved in water; or 3) vehicle (sterile water). The syringe was left in place for 5 minutes after the injection then withdrawn. Withdrawal was delayed to prevent drug solutions from leaking out the needle tract. After drug delivery and syringe withdrawal, wounds were closed in layers and animals placed in 37°C incubators overnight with food and water freely available. The following day, animals were returned to their cages with ad libitum access to food and water. Animals were then monitored daily to ensure good health. Four animals died during, or shortly after, surgery. These animals were replaced to yield final groups sizes of n = 8.

### Histological Preparation

48-hours after contusion injury animals were anesthetized (ketamine-xylazine) and transcardially perfused 0.5 ml of 2% lidocaine HCL, 0.1 ml of heparin, 300–500 ml of 0.9% NaCl and 500–1000 ml of 4% paraformaldehyde. Spinal cords were removed and post-fixed for 24 hrs then cryoprotected in 30% sucrose. A 10-mm block of spinal cord tissue, centered on the injection site, was cut into 6 serial sets of 20-um thick frontal sections using a cryostat and mounted on glass slides. To quantify tissue sparing, sections were stained with cresyl violet, for cell bodies, and Luxol fast blue, for myelin. For immunohistochemical labeling, sections were washed with PBS, permeabilized with 0.3% triton in PBS, and then incubated overnight with primary antibodies prepared in blocking solution. Neuron-Specific Nuclear Protein (NeuN; 1∶200 dilution; Chemicon, Temecula, CA, USA) is specific to neurons and in the spinal cord only labels cell in the gray matter [18], [19]. CC1 antibody (1∶500; Oncogene Research Products, Cambridge, MA) was used to identify oligodendrocytes. Primary antibodies were visualized through application of a secondary antibody for 1 hour at room temperature (Alexa-Fluor 488; Goat anti-Mouse IgG; 1∶250; Molecular Probes, Eugene, OR, USA). Sections were then rinsed with PBS and cover-slipped with Vectashield with DAPI (nuclear counterstain) hard-set mounting media (Vector's Laboratory Inc., Burlinghame, LA, USA).

### Anatomical Analysis

The center of the lesion (lesion epicenter) was identified as the area with maximal tissue damage. Tissue sparing was assessed at 1 mm-intervals centered on the lesion epicenter. Camera lucida drawings were made to quantify spared tissue and lesion areas. Lesion was defined as areas in the gray matter devoid of neuronal cells, areas absent of Luxol fast blue staining in the white matter, and/or areas of gliosis. The proportion of spared tissue area relative to total cross sectional area was calculated for each tissue section and averaged across 7 mm centered on the lesion epicenter.

The density of CC1 immunoreactivity in the dorsal column (DC) and ventral column (VC) white matter and the number of NeuN+ neurons were quantified through threshold-based measures of digital images with the MetaMorph Offline Meta Imaging Series version 6.3r software (Universal Imagining Systems, PA, USA). Images were captured for each hemi-cord at 1 mm intervals centered on the lesion epicenter using an Axioplan 2 imaging microscope (Zeiss, Oberkochen, Germany). Manual threshold levels were set to include positively stained cells while excluding background staining for each image. DC oligodendrocyte measurements were made using a standard size box aligned with medial edge of the dorsal horn of the gray matter and the dorsal edge of the spinal cord. VC oligodendrocyte measurements were made using a standard size box aligned with the ventral median sulcus and the ventral edge of the spinal cord. The proportional area of CC1+ immunoreactivity was recorded for each area. Neuron counts were estimated from the entire gray matter containing cells stained positively with NeuN. The length of the major axis of each cell was calculated using the integrated morphometry analysis function in metamorph in order to count small (10–15 µm in length), medium (15–30 µm in length), and large (over 30 µm in length) neurons. This definition was adapted from [20]. In that study, cresyl violet or hematoxylin-floxine labeled cells were counted in an automated fashion and were defined as small (5–10 µm), medium (10–15 µm), and large (>15 µm) according to the major axis. NeuN has a tendency to label dendrites and cell bodies along with the nucleus of the cell [19], so a larger size criteria was used to adapt this technique.

### Behavioral Analysis

Forelimb grooming function was assessed with a six-point scoring system as previously described [15]. Briefly, grooming activity was scored depending upon the area of the head contacted by the animal's forelimb: 0- no contact to any part of the face or head; 1-the underside of the chin and/or the mouth area; 2-the area between the nose and the eyes; 3- the eyes and/or the area up to the front of the ears; 4-the front but not the back of the ears; 5-area of the head behind the ears. Locomotor recovery was scored during open field observation for 4 minutes. Hindlimb function was assessed using the 13-point sub-scoring system for the Basso, Beattie, Bresnahan test of locomotor recovery [21], [22]. The sub-score system assigns values to each hindlimb for toe drags, paw position, tail position, and trunk instability (Table S1).

### Statistics

Open field and grooming data were analyzed using ANOVA. NeuN cell counts and all area measures were compared using a two-within, one-between mixed ANOVA. Within subject factors were side (ipsilateral or contralateral to the injury) and distance from the injection site; the between subject factor was treatment condition. Significant interactions were accounted for by examining either side (contralateral or ipsilateral) or distance (rostral or caudal) separately. Based on these interactions, neuron counts and sparing measures are reported only for the side ipsilateral to injury. ANOVA's were followed by Dunnett's or Tukey's test for multiple comparisons. Independent samples t tests were also used to compare two groups separately. Graphpad Grubb's test (extreme studentized deviate method http://www.graphpad.com/quickcalcs/Grubbs1.cfm) was used to remove statistical outliers. All p-values below 0.05 were considered significant. Unless noted otherwise, all data represent the mean ± SEM. All analyses were performed using SPSS 14 for Windows (SPSS Inc., Chicago, IL, USA) or GraphPad Prism 5.0 (GraphPad Software Inc., La Jolla, CA). Figures were prepared using Adobe Photoshop CS (Adobe Systems Inc., San Jose, CA) and GraphPad Prism 5.0.

## Results

There were no differences among vehicle, NBQX, or topiramate treated groups for any anatomical or functional outcome measures examined following moderate (6.25 mm) contusion injury (p>0.50 for ANOVAs of all outcome measures; data not shown). All the results below refer to treatment after severe (12.5 mm) contusion injury.

### Tissue Sparing

**Figure 1:**
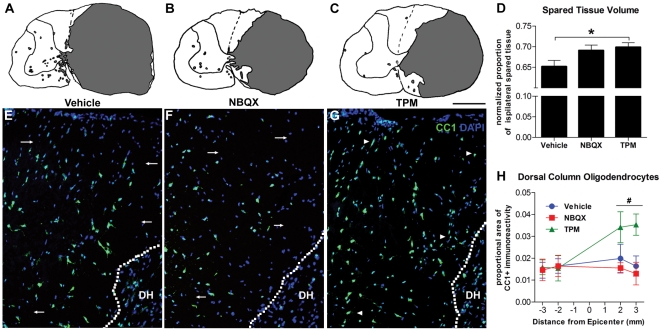
Tissue sparing and oligodendrocyte labeling 48 hrs after severe spinal cord injury. (A–D) Topiramate treated animals had more spared tissue compared to vehicle controls. (A–C) Reprehensive camera Lucida drawing of lesion epicenters from different treatment conditions (lesion area in gray). Notice the increased tissue sparing following topiramate (TPM) and NBQX treatment in the dorsal horn and ventromedial white matter compared to vehicle treated. D) Topiramate and NBQX-treatment increased the average proportional area of tissue spared. Area was quantified over 6 mm centered on the lesion epicenter. E–G) Representative examples of CC1 immunoreactivity in the dorsal columns caudal to the lesion epicenter. Arrows identify areas with no CC1+ cells in vehicle and NBQX conditions (E–F), while arrowheads (in G) identify the same areas with CC1+ cells in the topiramate condition. More CC1 labeling is also evident in the dorsal horn (DH) for topiramate treated animals. H) The density of CC1 immunoreactivity was significant increased with topiramate treatment. *p<0.05; p = 0.07 for NBQX vs. vehicle. ^#^p<0.05 for topiramate vs. vehicle or NBQX for average CC1 density caudal to the lesion epicenter.

Swelling occurs in the acutely injured spinal cord and can confound area based sparing measures [23]. To overcome this confound we normalized spared tissue areas to spinal cord cross sectional area. Animals receiving topiramate treatment 15 minutes after severe injury had significantly more spared tissue compared to vehicle treated controls (Fig. 1A–D; p<0.05). Sparing was measured 48 hrs after injury and primarily occurred in the dorsal medial and ventral medial white mater (Fig. 1A–C). NBQX-treatment increased sparing compared to controls but the effect was not statistically significant (p = 0.07, Fig. 1D).

### Oligodendrocyte Survival

We detected increased oligodendrocyte preservation in topiramate treated animals compared to vehicle or NBQX-treated controls (Fig. 1E–H). Topiramate treatment significantly improved oligodendrocyte sparing after severe SCI in the dorsal column white matter (p<0.05 vs. NBQX or vehicle; Fig. 1E) but not in the ventral white matter (p = 0.23; data not shown). No significant differences were detected between NBQX and vehicle control.

### Neuronal Survival

**Figure 2:**
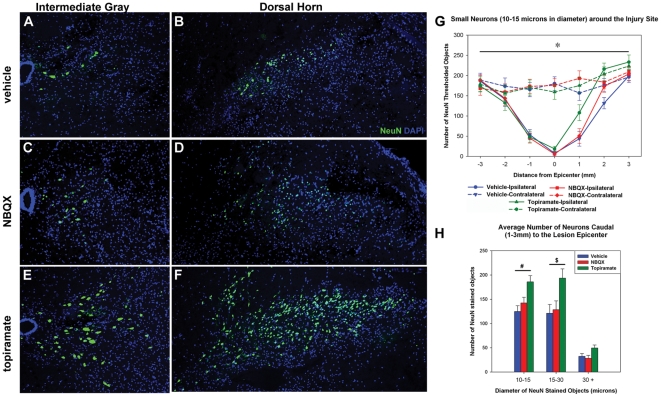
Topiramate treatment significantly increased NeuN+ neuron sparing 48-hrs after severe spinal cord injury. A–F) Representative examples of NeuN labeling ipsilateral to the side of injury 1 mm-caudal to lesion epicenter. Pictures are from around the central canal (A,C,E) and dorsal horn (B,D,F). Notice the increased number of neurons in the topiramate treated group. G) The number of small sized neurons remaining 48 hrs after injury by group. Graphs were similar for medium and large sized neurons. H) More small, medium, and large neurons were spared caudal to the lesion epicenter following topiramate treatment (ANOVA for large diameter neurons, p = 0.051). ^#,&^p<0.05 for topiramate vs. vehicle; ^#^p = 0.052 and ^&^0.057 for topiramate vs. NBQX. *There were significant differences among groups as a function of distance from the lesion epicenter; therefore only caudal neuron numbers were considered in (H) (p<0.05 for distance x group interaction two-way ANOVA).

Neurons are lost within the first 24 hrs following contusion injury [24]. In order to assess treatment-induced neuronal survival we counted the number of NeuN-positive cells 48 hrs after severe contusion injury. Animals receiving topiramate treatment had significantly more small and medium sized neurons compared to vehicle or NBQX-treated controls (Figure 2). Neurons were spared primarily in the dorsal horn and around the central canal of the gray matter (Fig. 2A–F). We also detected more large-diameter motoneurons sparing with topiramate treatment although the effect was not statistically significantly (ANOVA, p = 0.051; Fig. 2H). NBQX treatment did not improve motor neuron sparing compared to vehicle controls (Fig. 2).

### Behavioral Recovery

We assessed motor recovery by testing grooming and locomotor function 2 days after severe SCI. Grooming scores for animals treated with topiramate (1.3+/−0.3) were better then vehicle (1.0+/−0.3) or NBQX-treated (0.3+/−0.2), however significant effects were only detected for topiramate vs. NBQX-treated animals (p<0.05) (Fig. S1). There were no significant locomotor differences among treatment groups (Fig. S1). Animals had frequent to consistent weight supported stepping (>50% of the time) with some abnormal paw rotation, trunk instability, and tail drags.

## Discussion

Following the primary insult of spinal cord injury, toxic amounts of glutamate are released and large numbers of neurons and oligodendrocytes are lost through excitotoxic and other secondary mechanisms [3], [4]. We show that the AMPA receptor antagonist, topiramate, is effective at reducing this cell loss when delivered 15-minutes after SCI. Significantly more neurons and oligodendrocytes are spared in animals treated with topiramate after cervical spinal cord contusion injury compared to vehicle control. Topiramate is more effective than the well-documented neuroprotective AMPA receptor antagonist, NBQX. Unlike other glutamate receptor antagonists which have failed clinical trials due to undesired side effects [8], [10]; topiramate is clinically available for treatment of migraines and epileptic seizures and tolerated by individuals with SCI [25], [26]. To the best of the authors' knowledge, the current study is the first demonstration of topiramate's neuroprotective potential as a therapy for spinal cord injury.

A variety of factors contribute to neurotoxicity after SCI making it likely that combinatory approaches or multi-mechanistic drugs are needed for clinical efficacy. Mechanical induced depolarization causes cells to open voltage dependent K^+^, Na^+^, and Ca^2+^ channels and release excitatory neurotransmitters. Ultimately this increased activation causes intracellular Ca^2+^ accumulation and induces a cascade of molecular events resulting in cell death [6], [27]. While drugs with single mechanisms of action can be protective after injury, multi-mechanistic drugs may more effectively reduce excitoxic cell death. NBQX can improve mitochondrial function and reduced oxidative events after SCI [28]. Indeed, in agreement with of previous reports, we detected increased tissue sparing with NBQX-treatment vs. control (Fig. 1) [29]. Similar to other published reports, however, NBQX was not sufficient to reduce neuron loss in the current study (Fig. 2) [30].

The multi-mechanistic properties of topiramate make it a promising therapy for treating SCI. Topiramate is a broad spectrum anticonvulsant that in addition to antagonizing glutamate receptors, enhances the effects of the inhibitory neurotransmitter GABA; reduces the activity of voltage gated sodium and calcium channels; and blocks calcium influx into cells [26]. Since treatment with either GABA agonists or sodium channel blockers confers neuroprotection in models of SCI [31], [32], it is likely that these other mechanisms of action contribute to the increased neuroprotection detected with topiramate compared to NBQX.

In the current study we provide proof of principle evidence that topiramate can be neuroprotective when delivered after SCI and although we injected topiramate directly into the spinal cord, the dose used has clinical significance. We delivered a total of 30 nmols of topiramate into the cord. In rats, brain concentrations of topiramate reach roughly 1/1000 the concentration delivered orally [33]. The equivalent oral dose in the current study would be 40–50 mg/kg. Doses in this range lead to tissue sparing and behavioral improvements following damage to the CNS [11]–[13]; are below the value of ED_50_ causing motor impairments in rats [34]; and would likely be tolerated by humans [26].

Interestingly, topiramate was only effective when delivered after severe, but not moderate SCI. This is likely due to differences in secondary injury progression and overall damage between the two severities. Results of heparin treatment after cervical contusion injury indicate that microvasculature remains intact after milder injury [35]. Therefore less hemorrhage, a predictor of secondary injury and lesion progression [36], occurs with milder injury. Indeed, following cervical contusion injury, progressive gray matter loss is detectable only after severe, but not moderate or mild injury [37]. In addition, acute white matter loss, within 3 dpi, only occurs after severe injury [37]. Collectively, these finding predict the results of the current study; acute treatments aimed at limiting secondary injury progression after cervical injury, i.e. topiramate, may be more effective after severe injury.

Topiramate treatment improved oligodendrocyte sparing in the dorsal columns, but not ventral white matter. Topiramate was microinjected through the dorsal surface of the spinal cord. This route of administration may have resulted in less drug penetration into the ventral vs. dorsal spinal cord. Ventral column white matter is also further from impact site and myelinated axon loss is less pronounced here than in the dorsal columns after cervical injury [37]. Lack of penetration, both due to route of administration and maintained tissue integrity in the ventral columns, may therefore contribute to the region specific oligodendrocyte sparing following topiramate treatment.

Since topiramate treatment increased neuron sparing after SCI, it may be a useful drug to treat individuals with cervical injuries. In order for successful translation from basic science to clinical application, therapies should be tested in contusion models of injury and mimic treatment approaches applicable to humans [38]. The more accurately these models reproduce the human condition, the greater the potential for successful translation. We tested topiramate treatment in a model of cervical SCI. The cervical spinal cord is the most common spinal level of injury in humans and treatments that improve hand function are the number one priority of these individuals [39]. Treatments that spare motor neurons may address this priority. Indeed, neuronal sparing that reduces motor deficits by even one spinal level dramatically improves the quality of life for the cervically injured [40]. More work is needed to determine the therapeutic window of topiramate treatment and if treatment can increase long-term motor recovery. Nonetheless, by determining that topiramate is protective and spares neurons after severe, but not moderate injury, we now have insight into which injury populations may be suitable therapeutic targets.

We report that acute topiramate treatment is protective against secondary damage caused by SCI. In addition, repeated administration is tolerated in spinal cord injured patients, reduces injury induced pain in humans, and increases neurite outgrowth after nerve injury [25], [41]. Further studies are in progress to extend this potential therapy to preclinical treatment paradigms that are more relevant to clinical application, including systemic routes of administration and initiation of treatment at longer post-injury time points.

## Supporting Information

Figure S1 **Locomotor and grooming function 48 hrs after severe SCI.** (A) There were no significant effects of treatment on locomotor function. (B) Animals treated with NBQX had significant worse grooming function with the limb ipsilateral to injury compared to topiramate treated (*p<0.05). Neither NBQX nor topiramate treated animals differed significantly from vehicle. Scores for the limb contralateral to the injury did not differ from uninjured controls (data not shown).
(TIF)

Tables S1 Open field subscore catagories. IC = initial contact, LO =  lift off. Toe clearance and paw position are scored for each hindpaw individually then summed.
(DOC)
