# Supplementary figures and images for: Topiramate Treatment Is Neuroprotective and Reduces Oligodendrocyte Loss after Cervical Spinal Cord Injury
Source: PLoS One. 2012 Mar 13;7(3):e33519. doi: 10.1371/journal.pone.0033519 (PMC3302770; doi:10.1371/journal.pone.0033519)

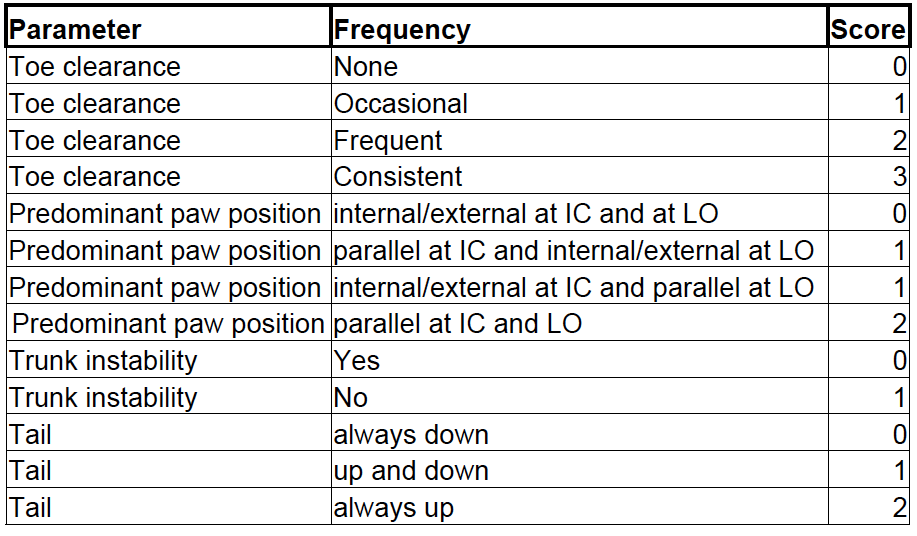

Supplement: Tables S1 — Open field subscore catagories. IC = initial contact, LO = lift off. Toe clearance and paw position are scored for each hindpaw individually then summed. (DOC) [file pone.0033519.s002.doc]
